# Bidirectional Associations Between Autonomic Function, Sleep Quality, and Daily Fatigue and Energy Symptoms in Long COVID: Longitudinal Digital Health Study

**DOI:** 10.2196/99630

**Published:** 2026-09-14

**Authors:** Nana Yaw Aboagye, Mark R Baker, Kenneth Baker, Silvia Del Din

**Affiliations:** 1 Translational and Clinical Research Institute, Faculty of Medical Sciences, Newcastle University, Newcastle upon Tyne, Newcastle, England, NE2 4HH, United Kingdom, +44 0191 2086897

**Keywords:** post-COVID fatigue symptoms, post-COVID-19 condition, heart rate variability, sleep quality, fatigue, wearable devices, exploratory longitudinal study

## Abstract

**Background:**

Long COVID is characterized by persistent fatigue, with disrupted autonomic function and sleep disturbances frequently reported. Whether daily symptom severity influences subsequent sleep and autonomic recovery (the reverse temporal direction) remains underexplored.

**Objective:**

This study aimed to examine bidirectional, within-person associations between objectively measured sleep quality, nocturnal heart rate variability (HRV), and daily fatigue and energy levels in individuals reporting post-COVID fatigue symptoms.

**Methods:**

We conducted a longitudinal observational study using continuous wearable monitoring (Fitbit Inspire 3) in 14 individuals with long COVID over a median of 28 days (range 12‐73 d), yielding 678,057 minute-level observations. Nocturnal HRV parameters (root mean square of successive differences [RMSSD], high-frequency power, low-frequency/high-frequency ratio) and sleep metrics (duration, efficiency, deep sleep, rapid eye movement [REM] sleep) were derived automatically. Fatigue (4-level scale) and energy (5-level scale, 0‐100) were reported via smartphone app (FatigueSense). Two temporal linking strategies were applied: (1) prospective, linking morning/afternoon symptom reports to the previous night’s sleep/HRV; and (2) reverse-direction, linking evening reports to the subsequent night’s sleep/HRV. Repeated-measures correlation (rmcorr) accounted for within-person clustering; person-mean–centered (Mundlak) cumulative link mixed models disaggregated within-person from between-person effects.

**Results:**

Prospective within-person rmcorr analyses (n=260 observations, 14 participants) identified 3 associations surviving Bonferroni correction (α=.0031): sleep duration (*r*=−0.276, 95% CI −0.392 to −0.152; *P*<.001) and REM sleep (*r*=−0.215, 95% CI −0.342 to −0.079; *P*=.002) with next-day fatigue, and sleep efficiency with next-day energy in the counterintuitive negative direction (*r*=−0.196, 95% CI −0.317 to −0.068; *P*=.003). HRV metrics showed no significant within-person day-to-day associations with symptoms (HRV-RMSSD → fatigue: *r*=−0.064, *P*=.36). Mundlak models revealed that HRV-RMSSD associations were predominantly between-person: individuals with chronically higher HRV reported consistently lower fatigue (β=−1.260, 95% CI −2.231 to −0.290; *P*=.01) and higher energy (β=0.539, 95% CI 0.056 to 1.022; *P*=.03). A significant Sleep × Steps interaction (β=0.091 per SD of steps per 1000; *P*=.02) indicated that the protective association of longer sleep on fatigue was attenuated on more active days, consistent with a postexertional malaise pattern. In an exploratory reverse-direction analysis, evening energy (but not fatigue) showed an association with that night’s HRV-RMSSD that was nominally significant but did not persist after Bonferroni correction (rmcorr: *r*=0.327, 95% CI 0.080 to 0.537; *P*=.01; α=.0050) and was supported by a log-linear mixed model (β=0.0069; *P*=.02).

**Conclusions:**

In this exploratory longitudinal study, sleep duration and REM sleep showed day-to-day (within-person) associations with next-day fatigue, while HRV-RMSSD distinguished individuals with better versus worse average symptom burden. The counterintuitive sleep efficiency-energy association most plausibly reflects chance, given the null sleep efficiency-fatigue association and the multiple-testing context. The preliminary, uncorrected reverse-direction association between evening energy and nocturnal HRV-RMSSD warrants replication. Experimental work is needed to test causal mechanisms and clinical relevance.

## Introduction

### Long COVID, Sleep, and Autonomic Dysfunction

Long COVID, or postacute sequelae of SARS-CoV-2 infection, affects many individuals following COVID-19 infection, resulting in persistent and often debilitating symptoms lasting months to years beyond the acute infection phase [1-4]. Among the most commonly reported symptoms are profound fatigue, postexertional malaise (PEM), and sleep disturbances, which collectively impair quality of life and functional capacity [5,6]. Understanding the physiological mechanisms underlying these symptoms and their temporal dynamics is critical for developing effective management strategies.

Sleep disturbances are prevalent in long COVID, with studies in post-COVID cohorts reporting sleep disruption in 40% to 75% of affected individuals [1,6]. Concurrently, disruptions in autonomic nervous system function have been documented, including reduced heart rate variability (HRV, a marker of parasympathetic activity and cardiovascular adaptability) [7,8]. Lower HRV has been associated with worse long COVID symptoms, suggesting impaired autonomic regulation may contribute to symptom persistence [9,10].

HRV, particularly during sleep when parasympathetic activity normally predominates, provides a window into autonomic recovery processes [11]. The high-frequency component of HRV (HRV-HF, 0.15‐0.4 Hz) reflects vagal/parasympathetic modulation, while the low-frequency component (HRV-LF, 0.04‐0.15 Hz) reflects mixed sympathetic and parasympathetic influences [12]. The LF/HF ratio is often interpreted as an index of sympathovagal balance, though its physiological specificity is debated [13]. In healthy individuals, sleep is characterized by stage-dependent autonomic modulation, with parasympathetic predominance during non–rapid eye movement (REM) sleep and relative sympathetic activation during REM [14].

### From Prediction to Bidirectional Association

Previous research has focused on unidirectional relationships, investigating how sleep quality or HRV predicts subsequent symptom severity [15]. A critical gap exists in understanding the reverse direction: whether daily symptom severity influences physiological recovery during subsequent sleep. This bidirectional perspective is particularly relevant in long COVID, where symptom-driven behavioral adaptations and psychological states may impact sleep quality and autonomic recovery [16].

### The Role of Physical Activity

PEM, a hallmark of long COVID and myalgic encephalomyelitis/chronic fatigue syndrome, involves symptom worsening following physical or cognitive exertion [17-19]. COVID-era physical inactivity has been documented as an additional concern for individuals recovering from SARS-CoV-2 infection [20]. The relationships between daily activity levels, sleep quality, and symptoms remain poorly understood. Evidence from accelerometer-based studies suggests that sleep duration, sedentary behavior, and physical activity should be considered as codependent daily behaviors rather than isolated exposures [21]. Activity may act as a moderator, with effects on symptoms depending on sleep quality.

### Study Aim

To address these gaps, we conducted an exploratory longitudinal study using continuous wearable device monitoring in individuals reporting post-COVID fatigue. We employed a bidirectional analytical approach examining (1) prospective associations: how does the previous night’s sleep/HRV relate to next-day symptoms?; (2) reverse-direction associations: how do evening symptoms relate to that night’s sleep/HRV?; and (3) activity moderation: does physical activity interact with sleep quality in predicting symptoms? We hypothesized that (H1) sleep duration would show within-person day-to-day associations with symptoms; (H2) evening symptom severity would predict subsequent nocturnal autonomic function; and (H3) physical activity would moderate the sleep-symptom relationship.

## Methods

### Study Design and Participants

This was a longitudinal observational study conducted between May 2025 and September 2025. Participants were recruited from individuals with post-COVID fatigue who had previously participated in a National Institute for Health and Care Research funded study at Newcastle University (Pausing-PCF [22]) and were engaged in patient and public involvement and engagement activities, as well as through online patient communities and social media. Inclusion was based on self-reported post-COVID fatigue symptoms and willingness to wear the device continuously. No formal clinician-adjudicated diagnosis was required, and no healthy control group was recruited; this represents an acknowledged limitation (see *Limitations*). This study uses a different cohort, device, and analytical framework from the authors’ prior feasibility study in this population [15], which involved 68 different participants, a different wearable device (Axivity AX6 accelerometer with VitalPatch ECG patch), and did not include reverse-direction associations or within-person effect disaggregation.

### Ethical Considerations

The study protocol was approved by the Newcastle University Ethics Committee (reference 47832‐2023). All procedures were conducted in accordance with relevant guidelines and regulations, and all participants provided written informed consent prior to enrollment. Participant data were deidentified prior to analysis. No individual identifying information appears in any figures or tables. Participants were volunteers who received no financial compensation. The ethics approval permitted secondary analysis of wearable data collected under this protocol without additional consent.

### Wearable Device Monitoring

Participants wore a Fitbit Inspire 3 wristband continuously (24 h/d) for the study duration, charging for approximately 1 to 2 hours per day during inactive periods (eg, while showering). The Inspire 3 uses photoplethysmography to estimate interbeat intervals, from which HRV metrics are derived using proprietary algorithms. It is important to note that validation studies of consumer wearable photoplethysmography sensors have primarily examined heart rate accuracy and sleep staging [23-25]. Where spectral HRV has been formally evaluated in ring- and wrist-worn photoplethysmography devices against electrocardiogram (ECG) during nocturnal monitoring, agreement has been inconsistent: nightly-average HF power shows moderate-to-good correspondence, but 5-minute segment estimates of LF power (*r*≈0.42) and LF/HF ratio (*r*≈0.36) show poor agreement with ECG-derived values [26,27]. No independent peer-reviewed study has specifically validated the Fitbit Inspire 3’s proprietary spectral HRV algorithm against ECG; these metrics should therefore be treated as indicative rather than definitive measures of autonomic function. The device’s internal HRV sampling algorithm processes photoplethysmography segments during sleep, enabling frequency-domain HRV estimation [28]; the “1 Hz” sampling figure sometimes quoted for this device refers to display resolution rather than the raw optical sampling rate, which is substantially higher. Participants were instructed that step data would be collected automatically during waking hours.

Sleep metrics extracted included sleep duration (min), sleep efficiency (proportion of time asleep while in bed, %), deep sleep duration (min), and REM sleep duration (min). The device automatically detected sleep onset, awakening, and sleep stages using movement patterns and heart rate data [29].

HRV metrics included HRV-RMSSD (root mean square of successive differences), a time-domain measure reflecting parasympathetic activity [30]; HRV-HF (high-frequency power, 0.15‐0.4 Hz), a frequency-domain measure of vagal modulation [12]; HRV-LF (low-frequency power, 0.04‐0.15 Hz); and the LF/HF ratio, included as an exploratory autonomic index but interpreted cautiously given its debated physiological specificity [13]. HRV metrics were measured exclusively during sleep, providing an index of nocturnal autonomic recovery. Sleep periods with <50% HRV coverage were excluded, as signal quality is lowest during restless sleep. Physical activity was quantified as daily step count accumulated between sleep offset and symptom report time (prospective analysis) or from sleep offset to evening report (reverse-direction analysis).

### Symptom Reporting

Participants completed brief symptom reports via the FatigueSense smartphone app [31] at their convenience throughout each day. Reports were automatically timestamped. Participants rated fatigue severity on a 4-level categorical scale (none, mild, moderate, severe); and energy level on a 5-level ordinal scale (0, 25, 50, 75, 100). Reports were categorized by time of day: morning (5 AM-11:59 AM); afternoon (noon-4:59 PM); evening (5 PM-9:59 PM); and night (10 PM-4:59 AM). Participants were encouraged to report at least once daily but were not assigned fixed reporting times, introducing potential variability in the gap between waking and morning report completion and between evening report and subsequent sleep onset.

### Temporal Linking Strategy

A key methodological feature of this study was the explicit use of symptom report timestamps to enable bidirectional analysis. Two analytically distinct datasets were created.

#### Prospective Linking (Sleep/HRV → Symptoms)

Morning and afternoon symptom reports were linked to the previous night’s sleep and HRV data. For each report, we extracted sleep metrics from the most recent completed sleep period, HRV metrics averaged across that sleep period, and step count accumulated between sleep offset and symptom report time. This addresses the question: “How does last night’s sleep and autonomic activity predict how I feel today?”

#### Reverse-Direction Linking (Symptoms → Sleep/HRV)

Evening symptom reports were linked to the subsequent night’s sleep/HRV (the sleep period beginning after the evening report). Step count for this dataset was accumulated from previous sleep offset to evening report time. This addresses the question: “How is tonight’s sleep and autonomic recovery associated with how I felt this evening?” Note that this direction is temporally prospective (symptoms precede the sleep period); we use “reverse-direction” to distinguish it from the prospective symptom-prediction analysis.

### Data Preprocessing

Data were extracted via the Fitbit Web API. HRV metrics (RMSSD, HF power, and LF power) were obtained from the intraday HRV end point (fields hrvRmssd, hrvHf, and hrvLf), which returns values at a native 5-minute resolution during device-detected sleep; the device does not expose beat-to-beat interbeat intervals through this endpoint. For each sleep period, HRV metrics were averaged across all available 5-minute segments, and periods with <50% segment coverage were excluded. Across the cohort, 36,521 five-minute HRV segments were recorded over 471 sleep periods (mean ≈78 segments, ≈6.5 h per period); thus, the “HRV segments” we report denote counts of 5-minute windows rather than individual minute-level readings. Sleep periods were identified using device-detected onset and offset times. Daily aggregates were computed for each 24-hour period (noon to 11:59 AM the following day). Symptom reports were matched to the appropriate sleep period based on timestamps.

### Statistical Analysis

All analyses were conducted in Python 3.13 and R (version 4.5.2; R Foundation for Statistical Computing) using the ordinal, lme4, lmerTest, repeated-measures correlation (rmcorr), and car packages.

#### Descriptive Statistics

Summary statistics were computed for all variables. All continuous variables showed significant departures from normality (Shapiro-Wilk test, *P*<.05), informing the choice of methods. Fatigue was a 4-level ordinal scale; no participants reported “None,” so 3 categories were observed (mild, moderate, severe). Energy was a 5-level ordinal scale; participants rarely selected 0 or 100. Restricted variance in both scales represents a measurement limitation (see *Limitations*).

#### Observation Flow and Sample Sizes

Of 260 prospective observations linked to a prior sleep period, 222 had complete HRV data (RMSSD+LF/HF). Complete-case counts differed by one observation between outcomes because of a single missing energy rating: the parsimonious cumulative link mixed models (CLMMs) used 215 (fatigue) and 214 (energy) observations, and the comprehensive and Mundlak CLMMs used 211 (fatigue) and 210 (energy). Of 105 reverse-direction observations, 71 had complete HRV data for the reverse-direction model. A total of (n=243, 93.5%) prospective observations had step data. Intraclass correlation coefficients (ICCs) were estimated from null CLMMs: fatigue ICC=0.245 (24.5% between-person variance); energy ICC=0.491 (49.1% between-person variance). The high ICC for energy indicates that between-person differences are substantial, necessitating disaggregation of within- and between-person effects.

#### Bivariate Associations: rmcorr

Bivariate associations were assessed using rmcorr [32], which accounts for within-person clustering by partialling out participant-level mean differences. This approach avoids the pseudoreplication error that occurs when pooling repeated observations from the same individuals and treating them as independent. Pooled Spearman correlations are presented in Multimedia Appendix 1 for comparison, but rmcorr estimates are the primary bivariate results. Statistical significance was assessed at α=.05 with Bonferroni correction within each domain (prospective: 16 tests, α_corrected_=.0031; reverse-direction: 10 tests, α_corrected_=.0050).

#### Mixed-Effects Models

##### Prospective Models (Mundlak Specification)

Given the substantial between-person variance, particularly for energy (ICC=0.491), we employed person-mean–centered (Mundlak) CLMMs to disaggregate within-person (day-to-day) from between-person effects [33]. For each predictor X, we computed the person mean (between-person effect, hereafter “bp_”) and the deviation from the person mean (within-person effect, hereafter “wp_”). Both were included as separate predictors in the CLMM. This allows the within-person coefficient (the primary scientific question: “Does your HRV tonight predict your symptoms tomorrow compared to your own average?”) to be estimated independently of stable between-person differences.

Standard CLMMs (without mean-centering) were also fitted as a sensitivity analysis, using parsimonious (sleep duration+HRV-RMSSD+LF/HF ratio) and comprehensive (all sleep metrics+HRV metrics) specifications. Random intercept-only models were used throughout; likelihood ratio tests confirmed that random slopes did not improve fit (fatigue: likelihood ratio *P*=.52; energy: likelihood ratio *P*=.87).

Variance inflation factors (VIFs) were computed for the comprehensive model to assess collinearity. All VIFs were <2 (range 1.10‐1.84), indicating no meaningful multicollinearity.

All continuous predictors were *z* score standardized (mean 0, SD 1) to enable effect size comparison via standardized coefficients. 95% CIs are reported for all model estimates.

##### Reverse-Direction Model

A linear mixed-effects model (lmer) was fitted predicting log-transformed nocturnal HRV-RMSSD from evening fatigue and energy, with random intercepts for participants. Log transformation was applied because HRV-RMSSD is right-skewed; Shapiro-Wilk tests confirmed substantially improved residual normality under log transformation (raw: W=0.646, *P*<.001; log: W=0.941, *P*=.002), although some departure remained, which is noted as a limitation. The coefficient for energy is back-transformed to a percentage change per 25-point energy increase. We note that energy is modeled as an ordinal outcome in the CLMMs but enters this reverse-direction model as a continuous predictor of HRV-RMSSD. This continuous treatment is a deliberate, justified simplification: the 5 energy levels are anchored at equally spaced numeric values (0, 25, 50, 75, 100), so treating them as interval-scaled for use as a predictor is reasonable and yields the interpretable “per 25-point increase” effect; the ordinal treatment is retained where energy is the outcome because the proportional-odds model makes no equal-spacing assumption. The 2 treatments are therefore applied consistently to each role (energy-as-outcome vs energy-as-predictor) rather than inconsistently to the same quantity.

### Activity Pattern Analysis

#### Activity Tertiles

Participants were stratified into low, medium, and high activity groups based on step count tertiles. Mean fatigue and energy were compared across groups using Kruskal-Wallis tests with pairwise comparisons.

#### PEM and Sleep × Steps Interaction

To formally test whether physical activity moderated the sleep-symptom relationship, a linear mixed-effects model was fitted predicting fatigue (numeric) from sleep duration (*z* scored), steps per 1000 (*z* scored), and their interaction (sleep duration × steps/1000). Steps were rescaled to per-1000 units to yield interpretable coefficients. This continuous interaction model tests whether the association between longer sleep and lower fatigue differs depending on activity level.

## Results

### Sample Characteristics

Fourteen individuals reporting post-COVID fatigue symptoms (12 female [85.7%], 2 male [14.3%]; age range 35‐64 y) enrolled and completed the study protocol. Age distribution: 35 to 44 years (n=4, 28.6%), 45‐54 years (n=3, 21.4%), 55‐64 years (n=7, 50%). Participant characteristics and monitoring details are summarized in Table 1. Descriptive statistics for all variables are presented in Table 2.

**Table 1.** Participant and study characteristics (N=14).

Characteristic	Value
Sex, n (%)	
Female	12 (85.7)
Male	2 (14.3)
Age range (y)	35‐64
35‐44, n (%)	4 (28.6)
45‐54, n (%)	3 (21.4)
55‐64, n (%)	7 (50)
Monitoring duration	
Median days worn (range)	28 (12‐73)
Total minute-level observations	678,057
Total sleep periods	471
Total HRV^a^ 5-minute segments recorded	36,521
Mean HRV coverage per sleep period, % (SD)	77.6 (29.4)
Symptom reports (N=598), n (%)	
Morning reports (5 AM-11:59 AM)	261 (43.6)
Afternoon reports (noon-4:59 PM)	83 (13.9)
Evening reports (5 PM-9:59 PM)	204 (34.1)
Night reports (10 PM-4:59 AM)	50 (8.4)
Analytical datasets	
Prospective observations, raw (with complete HRV)	260 (222)
Complete data: parsimonious CLMM^b^ (fatigue/energy)	215/214
Complete data: comprehensive and Mundlak CLMM (fatigue/energy)	211/210
Observations with step data, n (%)	243 (93.5)
Participants in prospective analysis	14
Reverse-direction observations, raw (with complete HRV)	105 (71)
Participants in reverse-direction analysis	12
Fatigue ICC^c^ (null CLMM)	0.245
Energy ICC (null CLMM)	0.491

a HRV: heart rate variability.

b CLMM: cumulative link mixed models.

c ICC: intraclass correlation coefficients.

**Table 2.** Descriptive statistics for sleep, heart rate variability, activity, and symptom variables^a^.

Variable	Values
Sleep metrics, mean (SD)	
Sleep duration, min	470 (103)
Sleep efficiency, %	90.2 (9.9)
Deep sleep, min	65 (26)
REM^b^ sleep, min	84 (33)
HRV^c^ metrics (nocturnal), mean (SD)	
HRV-RMSSD^d^, ms	34.0 (23.3)
HRV-HF^e^, ms^2^	495 (1337)
HRV-LF^f^, ms^2^	1181 (2057)
LF/HF ratio	4.51 (3.08)
Physical activity, mean (SD)	
Daily steps (waking window)	7593 (3806)
Symptoms, n (%)	
Fatigue	
Mild	170 (28.5)
Moderate	388 (65.0)
Severe	39 (6.5)
Energy	
25	141 (23.7)
50	336 (56.4)
75	117 (19.6)
100	2 (0.3)

a HRV metrics were measured during sleep only. Daily steps reflect waking-window accumulation between sleep offset and symptom report time. All continuous variables showed significant departures from normality (Shapiro-Wilk test, *P*<.05). Sleep, HRV and activity statistics are computed over the 260 linked prospective observations; symptom counts are over all reports. Symptom percentages are of reports with a valid rating for that item (fatigue n=597; energy n=596, of 598 total reports).

b REM: rapid eye movement.

c HRV: heart rate variability.

d RMSSD: root mean square of successive differences.

e HF: high-frequency.

f LF: low-frequency.

### Prospective Associations: Sleep and HRV Predicting Next-Day Symptoms

**Figure 1.**
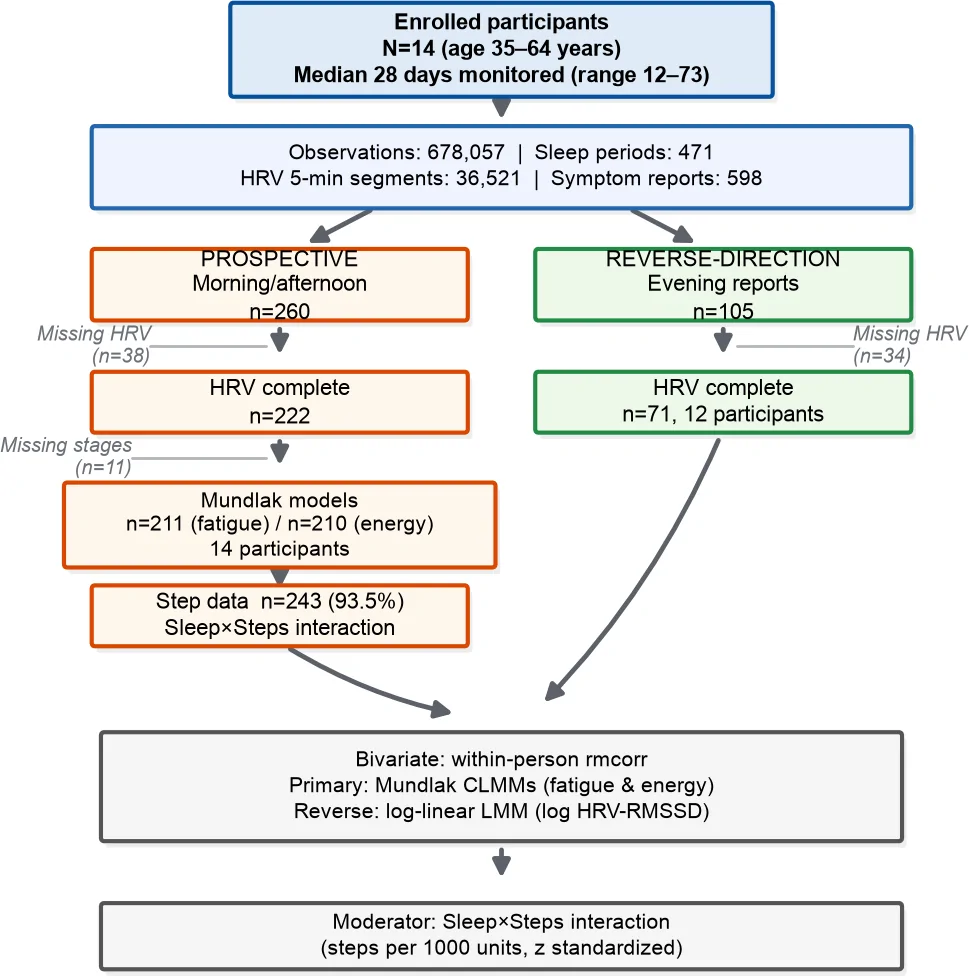
Observation and analysis flow diagram. CLMM: cumulative link mixed model; HRV: heart rate variability; LMM: linear mixed model; rmcorr: repeated-measures correlation; RMSSD: root mean square of successive differences.

We examined how the previous night’s sleep and HRV were associated with next-day fatigue and energy (n=260 prospective observations, 14 participants). Figure 1 illustrates the observation flow.

#### Within-Person Bivariate Associations (rmcorr)

**Figure 2.**
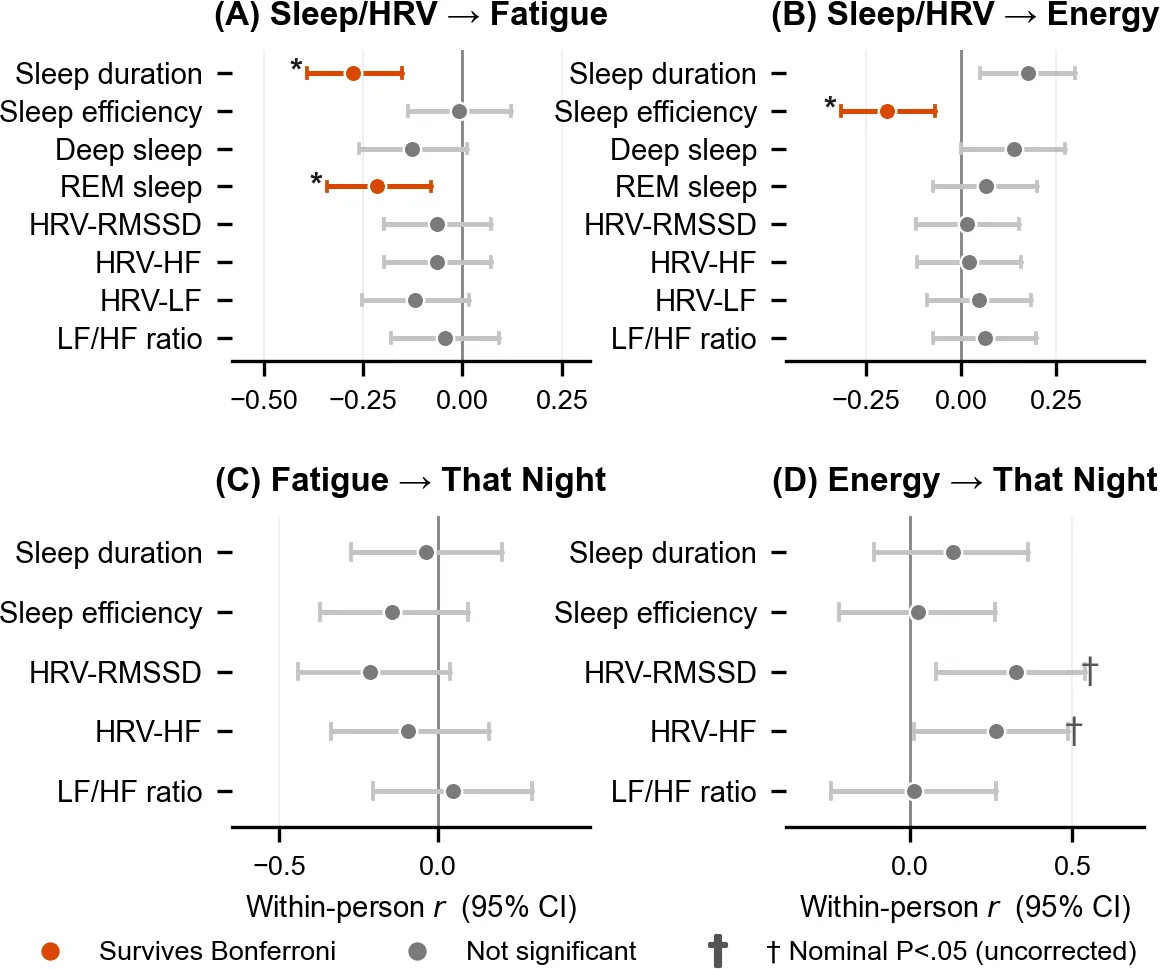
Bidirectional within-person associations (repeated-measures correlation), with points representing within-person *r* with their 95% CI. CLMM: cumulative link mixed model; HF: high-frequency; HRV: heart rate variability; LF: low-frequency; REM: rapid eye movement; RMSSD: root mean square of successive differences.

Within-person rmcorrs are presented in Table 3 and Figure 2.

**Table 3.** Prospective within-person associations: repeated-measures correlations^a^.

Predictor	Fatigue, *r* (95% CI)	*P* value	Energy, *r* (95% CI)	*P* value
Sleep duration	−0.276 (−0.392 to −0.152)	<.001^b^	0.176 (0.048 to 0.299)	.007
Sleep efficiency	−0.008 (−0.137 to 0.122)	.91	−0.196 (−0.317 to −0.068)	.003^b^
Deep sleep	−0.127 (−0.261 to 0.012)	.07	0.138 (−0.001 to 0.272)	.05
REM^c^ sleep	−0.215 (−0.342 to −0.079)	.002^b^	0.064 (−0.075 to 0.200)	.37
HRV^d^-RMSSD^e^	−0.064 (−0.198 to 0.072)	.36	0.016 (−0.120 to 0.152)	.81
HRV-HF^f^	−0.064 (−0.198 to 0.072)	.36	0.020 (−0.116 to 0.156)	.77
HRV-LF^g^	−0.120 (−0.252 to 0.016)	.08	0.047 (−0.090 to 0.182)	.50
LF/HF ratio	−0.044 (−0.179 to 0.092)	.53	0.063 (−0.074 to 0.197)	.37

a Bonferroni-corrected threshold for 16 tests: α=.0031.

b Survives Bonferroni correction (*P*<.003). Correlations are within-person (day-to-day) estimates using the rmcorr package; they reflect associations net of stable between-person differences. Analytic N varies by predictor owing to missing data (fatigue: 214‐243; energy: 213‐242); participants n=14.

c REM: rapid eye movement.

d HRV: heart rate variability.

e RMSSD: root mean square of successive differences.

f HF: high-frequency.

g LF: low-frequency.

After Bonferroni correction (α=.0031, 16 tests), 3 within-person associations survived: sleep duration was negatively associated with next-day fatigue (*r*=−0.276, 95% CI −0.392 to −0.152; *P*<.001); REM sleep was negatively associated with fatigue (*r*=−0.215, 95% CI −0.342 to −0.079; *P*=.002); and sleep efficiency was negatively associated with energy (*r*=−0.196, 95% CI −0.317 to −0.068; *P*=.003). The direction of this last association was counterintuitive, in that higher sleep efficiency was associated with lower next-day energy. Given that sleep efficiency showed no association with next-day fatigue (*r*=−0.008, *P*=.91) and the number of tests conducted, it is most plausibly a chance finding and requires replication. Sleep duration was also positively associated with next-day energy (*r*=0.176, 95% CI 0.048 to 0.299; *P*=.007), and deep sleep showed marginal associations with both fatigue (*r*=−0.127, *P*=.07) and energy (*r*=0.138, *P*=.05); none of these survived Bonferroni correction.

Notably, no HRV metric showed a significant within-person day-to-day association with next-day fatigue or energy after correcting for multiple comparisons (HRV-RMSSD → fatigue: *r*=−0.064, 95% CI −0.198 to 0.072; *P*=.36; HRV-RMSSD → energy: *r*=0.016, 95% CI −0.120 to 0.152; *P*=.81; HRV-HF → fatigue: *r*=−0.064, *P*=.36; HRV-HF → energy: *r*=0.020, *P*=.77). These within-person results contrast with pooled Spearman correlations (presented in Multimedia Appendix 1), which showed associations of ρ=−0.317 (HRV-RMSSD → fatigue) and ρ=0.416 (HRV-RMSSD → energy). The discrepancy reflects inflation of pooled correlations by stable between-person differences in HRV and symptom burden (ICC_energy=0.491), and underscores the importance of within-person analysis for repeated-measures data.

#### Person-Mean–Centered Mixed-Effects Models (Mundlak)

**Figure 3.**
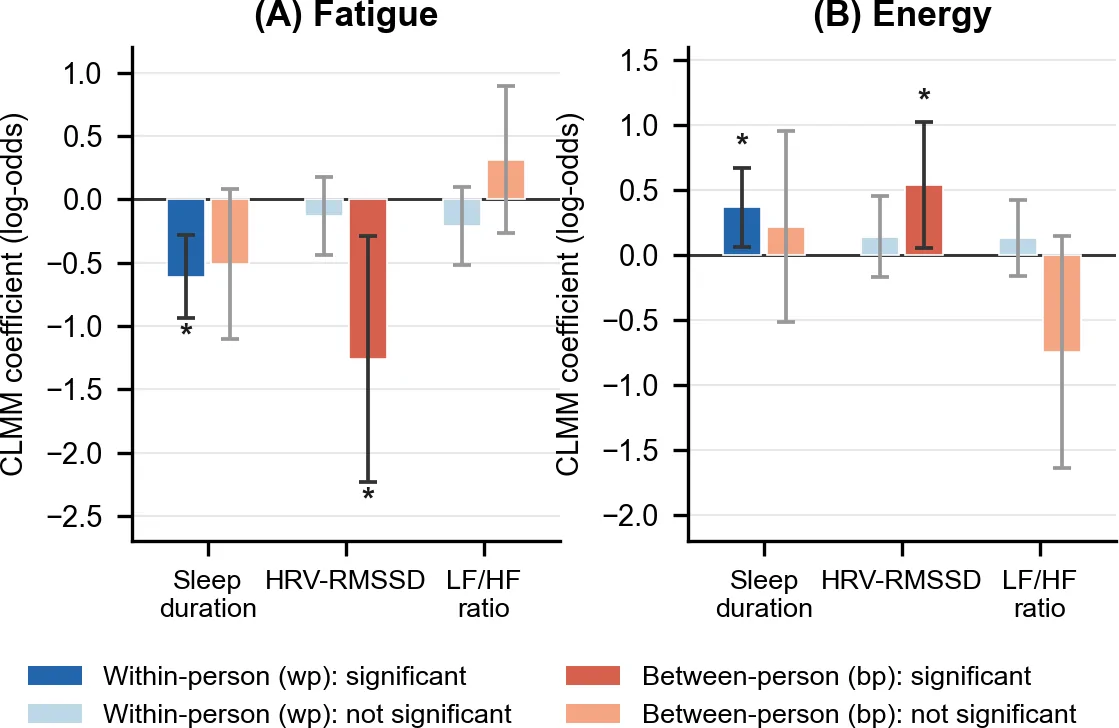
Within-person versus between-person effects (Mundlak cumulative link mixed models; n=211 [fatigue]/n=210 [energy], 14 participants). bp: between-person mean; CLMM: cumulative link mixed model; HF: high-frequency; HRV: heart rate variability; LF: low-frequency; RMSSD: root mean square of successive differences; wp: within-person deviation. *P<.05.

Mundlak CLMMs disaggregating within-person from between-person effects are presented in Table 4 and visualized in Figure 3.

**Table 4.** Person-mean–centered (Mundlak) cumulative link mixed models: within-person and between-person effects on fatigue and energy^a^.

Effect	Fatigue, β (95% CI)	*P* value	Energy, β (95% CI)	*P* value
Within-person (wp_)^b^				
wp_sleep duration	−0.610 (−0.937 to −0.283)	<.001	0.368 (0.066 to 0.671)	.02
wp_HRV^c^-RMSSD^d^	−0.130 (−0.437 to 0.177)	.41	0.144 (−0.166 to 0.453)	.36
wp_LF^e^/HF^f^	−0.215 (−0.523 to 0.093)	.17	0.135 (−0.158 to 0.427)	.37
Between-person (bp_)^g^				
bp_sleep duration	−0.509 (−1.101 to 0.083)	.09	0.220 (−0.517 to 0.958)	.56
bp_HRV-RMSSD	−1.260 (−2.231 to −0.290)	.01	0.539 (0.056 to 1.022)	.03
bp_LF/HF	0.313 (−0.267 to 0.893)	.29	−0.746 (−1.637 to 0.145)	.10

a All predictors *z* score standardized (mean 0, SD 1). Random intercepts for participants. intraclass correlation coefficient_fatigue=0.245; intraclass correlation coefficient_energy=0.491.

b wp_ : within-person deviation from own mean.

c HRV: heart rate variability.

d RMSSD: root mean square of successive differences.

e LF: low-frequency.

f HF: high-frequency.

g bp_ : between-person mean.

The analyses included 211 observations for fatigue and 210 observations for energy from 14 participants. The energy model had 1 fewer complete case than the fatigue model, consistent with the parsimonious and comprehensive CLMMs (Tables S1 and S2 in Multimedia Appendix 1).

For fatigue, within-person (day-to-day) sleep duration was a significant predictor (wp_sleep duration: β=−0.610, 95% CI −0.937 to −0.283; *P*<.001), indicating that on nights with longer sleep than a participant’s own average, next-day fatigue tended to be lower. The within-person HRV-RMSSD effect was not significant (wp_hrv_rmssd: β=−0.130, 95% CI −0.437 to 0.177; *P*=.41). However, the between-person HRV-RMSSD effect was significant (bp_hrv_rmssd: β=−1.260, 95% CI −2.231 to −0.290; *P*=.01), indicating that participants with chronically higher mean HRV-RMSSD reported consistently lower fatigue.

For energy, within-person sleep duration was again a significant predictor (wp_sleep duration: β=0.368, 95% CI 0.066 to 0.671; *P*=.02). The within-person HRV-RMSSD effect was not significant (wp_hrv_rmssd: β=0.144, 95% CI −0.166 to 0.453; *P*=.36), while the between-person effect was significant (bp_hrv_rmssd: β=0.539, 95% CI 0.056 to 1.022; *P*=.03).

VIFs for all predictors ranged from 1.10 to 1.84, confirming no problematic collinearity.

#### Sensitivity Analyses: Standard CLMMs

For comparison, standard CLMMs are presented in Multimedia Appendix 1 (Tables S1 and S2). In the parsimonious model (n=215 for fatigue, n=214 for energy), sleep duration (fatigue: β=−0.702, *P*<.001; energy: β=0.451, *P*=.006) and HRV-RMSSD (fatigue: β=−0.492, *P*=.05; energy: β=0.613, *P*=.009) were both significant. In the comprehensive model (n=211), only HRV-RMSSD remained significant (fatigue: β=−0.705, 95% CI −1.324 to −0.086, *P*=.03; energy: β=0.546, 95% CI 0.104 to 0.988, *P*=.02). These results should be interpreted with caution because the standard CLMMs do not disaggregate within-person from between-person effects, and with energy ICC=0.491 the estimates reflect a mix of both. Primary analysis was therefore based on the Mundlak models.

### Reverse-Direction Associations: Evening Symptoms Predicting Nocturnal HRV

We examined whether evening symptom severity predicted that night’s sleep and HRV (n=105 reverse-direction observations, 71 with complete HRV, 12 participants). These results are presented in Table 5.

**Table 5.** Reverse-direction within-person associations: evening symptoms predicting same-night sleep/heart rate variability^a^.

Outcome	Fatigue, *r* (95% CI)	*P* value	Energy, *r* (95% CI)	*P* value
Sleep duration	−0.037 (−0.273 to 0.203)	.77	0.134 (−0.110 to 0.362)	.28
Sleep efficiency	−0.146 (−0.371 to 0.096)	.24	0.024 (−0.217 to 0.263)	.85
HRV^b^-RMSSD^c^	−0.215 (−0.443 to 0.039)	.10	0.327 (0.080 to 0.537)	.01^d^
HRV-HF^e^	−0.094 (−0.337 to 0.162)	.47	0.265 (0.012 to 0.486)	.04^d^
LF^f^/HF ratio	0.048 (−0.206 to 0.297)	.71	0.013 (−0.242 to 0.266)	.92

a Bonferroni-corrected threshold for 10 tests: α=.0050.

b HRV: heart rate variability.

c RMSSD: root mean square of successive differences.

d Nominally significant (*P*<.05) but does not survive Bonferroni correction (α=.0050); no reverse-direction bivariate association survived correction. The evening energy → HRV-RMSSD association is independently supported by the log-linear mixed model (β=0.0069, *P*=.02). Analytic N varies by outcome owing to missing data (fatigue: 72‐81; energy: 71‐80); HRV outcomes include 12 participants, sleep outcomes 14. rmcorr within-person estimates.

e HF: high-frequency.

f LF: low-frequency.

#### Within-Person Bivariate Associations (rmcorr)

Higher evening energy showed a nominally significant positive within-person association with that night’s HRV-RMSSD (*r*=0.327, 95% CI 0.080 to 0.537; *P*=.01), though this did not survive Bonferroni correction (α_corrected=.0050, 10 tests). Neither HRV-HF (*r*=0.265, *P*=.04) nor any other reverse-direction bivariate association survived correction. Evening fatigue showed no significant associations with any sleep or HRV outcome (HRV-RMSSD: *r*=−0.215, *P*=.10; all others *P*>.20). Neither symptom predicted sleep duration or efficiency.

#### Log-Linear Mixed Model

A log-linear mixed-effects model (predicting log [HRV-RMSSD] from evening fatigue and energy) was consistent with the within-person association: evening energy independently predicted that night’s log-transformed HRV-RMSSD (β=0.0069, 95% CI 0.0013 to 0.0125; *P*=.02), while fatigue did not (β=−0.029, *P*=.69). Each 25-point increase in evening energy (on the 0‐100 scale) was associated with approximately 18.8% higher nocturnal HRV-RMSSD (ratio: 1.188, corresponding to approximately 6.4 ms at the mean level of 34.0 ms). As this association did not survive Bonferroni correction in the bivariate analysis, it is interpreted as exploratory (see *Discussion*).

Residual normality under log transformation (Shapiro-Wilk *W*=0.941, *P*=.002) was substantially better than the raw model (*W*=0.646, *P*<.001), supporting the log-transformed approach, although some departure from normality remained.

### Physical Activity Patterns and Symptom Interactions

#### Activity Tertiles

Activity tertile thresholds were low (<5684 steps), medium (5684‐8545 steps), and high (>8545 steps). Mean fatigue and energy levels showed minimal differences across tertiles (low: fatigue=1.83, energy=51; medium: fatigue=1.78, energy=47; high: fatigue=1.72, energy =50). Kruskal-Wallis tests confirmed these differences were not statistically significant (fatigue: χ²_2_=1.102, *P*=.58; energy: χ²_2_=1.879, *P*=.39).

#### Sleep × Steps Interaction

To formally test whether activity moderated the sleep-symptom relationship, a linear mixed-effects interaction model was fitted. Sleep duration (*z* scored) and steps per 1000 (*z* scored) were included as main effects along with their interaction:

fatigue_num ~ sleep duration_*z* × steps_per 1000_*z* + (1 | participant)

The Sleep × Steps interaction was significant (β=0.091, 95% CI 0.018 to 0.164; *P*=.02). The positive coefficient indicates that the protective association of longer sleep on fatigue was weaker (attenuated) at higher activity levels. This pattern is consistent with a PEM mechanism: on more active days, the symptom-protective effect of sleep may be partially offset by activity-related physiological burden. Step count alone was not significantly associated with fatigue (β=−0.056, *P*=.09) or energy (tertile analysis, *P*>.39), indicating no simple linear activity-symptom relationship.

Step data were available for 93.5% (243/260) of prospective observations. This figure represents the proportion of waking windows between sleep offset and symptom report in which at least some step activity was recorded. This differs from the proportion of all monitored waking-period time with continuous step activity, which was lower due to sedentary periods.

## Discussion

### Summary of Main Findings

This exploratory longitudinal wearable study examined bidirectional associations between sleep, nocturnal autonomic function, and daily symptoms in individuals with long COVID. The main findings were as follows: (1) sleep duration and REM sleep showed significant within-person (day-to-day) associations with next-day fatigue, and sleep efficiency showed a counterintuitive negative association with next-day energy that is most plausibly a chance finding; (2) HRV-RMSSD was a significant between-person predictor; individuals with chronically higher HRV reported consistently lower fatigue and higher energy but did not show significant within-person day-to-day associations; (3) physical activity interacted with sleep duration, with the protective effect of sleep on fatigue attenuated on more active days; and (4) in an exploratory reverse-direction analysis, evening energy (but not fatigue) showed a preliminary association with that night’s nocturnal HRV that did not survive Bonferroni correction and requires replication.

### Within-Person Sleep Effects and Between-Person HRV Associations

The distinction between within-person and between-person effects is conceptually critical for this study. The Mundlak models and rmcorr analyses converge on a consistent picture: day-to-day fluctuations in sleep duration predict day-to-day fluctuations in fatigue within the same individual. This is the most directly actionable finding: on nights when a person with long COVID sleeps longer than usual, they tend to feel less fatigued the next day.

The HRV-RMSSD finding is different in nature. Individuals with chronically higher HRV tend to report chronically better symptoms, but a given night's HRV-RMSSD does not predict whether that same individual will have a better or worse day of symptoms than their own average. This between-person difference may reflect stable individual characteristics, including underlying autonomic regulation capacity, severity of long COVID–related dysautonomia [34,35], baseline cardiovascular fitness, or other constitutional factors. This finding does not support HRV monitoring as a real-time, day-to-day symptom predictor in this population but does position HRV as a potentially useful biomarker of individual physiological reserve and overall symptom burden.

The pooled Spearman correlations (Multimedia Appendix 1) appeared to show much stronger HRV-symptom associations (ρ=−0.317 to −0.359 for fatigue, ρ=0.416 to 0.498 for energy). These were inflated by between-person variance: participants with higher average HRV also had lower average symptom burden, and, by treating 260 clustered observations as independent, this inflated both correlation coefficients and *P* values. The rmcorr and Mundlak analyses are methodologically appropriate for addressing the within-person scientific question and should be considered the primary analysis.

### Exploratory Reverse-Direction Association

In an exploratory analysis of the reverse-direction pathway, evening energy but not fatigue showed a within-person association with that night’s HRV-RMSSD (rmcorr *r*=0.327, *P*=.01; log-linear model β=0.0069, *P*=.02). This association was nominally significant but did not survive Bonferroni correction (α=.0050) and is therefore preliminary. If replicated, it would be consistent with the hypothesis that energy and fatigue reflect partially distinct processes: energy, assessed on a 0 to 100 scale, may capture perceived physiological vitality more sensitively than the 4-level fatigue scale, which was dominated by “moderate” responses (388/597, 65% of reports) and whose restricted variance may have limited its sensitivity (see *Limitations*). Energy levels on this scale cannot be equated with physiological capacity, and the association may partly reflect shared diurnal patterning, the 24-hour movement behavior framework [21], or other unmeasured factors. Replication in larger, adequately powered samples is required before any inference is drawn.

### Physical Activity: Context-Dependent Effects

Neither activity level alone nor its direct association with symptoms was significant. However, the significant Sleep × Steps interaction (*P*=.02) suggests that the relationship between sleep and fatigue is context-dependent. On more active days, longer sleep conferred less symptomatic benefit than on less active days. This is consistent with PEM mechanisms [36], wherein greater exertion may place demands on physiological recovery that partially counteract the restorative function of sleep, and with behavioral evidence that perceived effort shapes activity decisions in individuals with fatigue [37]. These findings align with emerging evidence that sleep, sedentary time, and physical activity should be considered as co-dependent daily behaviors influencing symptom outcomes [21]. The findings do not support direct exercise prescription, particularly given PEM concerns in this population. Low-burden, symptom-contingent movement approaches, including mind-body modalities such as Tai Chi, Qigong, or yoga, may offer more suitable future intervention directions [38-40], though the evidence specifically in long COVID requires dedicated investigation.

Activity guidance in long COVID may need to be personalized according to recent sleep quality and daily energy levels, in line with adaptive pacing principles [41].

### Methodological Contributions

A key contribution of this study is the explicit use of symptom report timestamps to construct distinct prospective and reverse-direction analytical datasets, enabling temporally grounded bidirectional analysis. The use of rmcorr and Mundlak CLMMs represents an advance over standard pooled analyses and random-intercept-only models in repeated-measures ecological momentary assessment research.

Future studies in this space should preregister the distinction between within-person and between-person hypotheses, use rmcorr or mixed-model–based estimates as primary bivariate descriptors, and ideally include Mundlak specifications in all primary analyses.

### Limitations

Several limitations should be considered when interpreting these findings. First, the small sample size (N=14) limits statistical power and the generalizability of the results. The cohort was predominantly female (n=12, 85.7%) and largely aged 45 to 64 years, reducing applicability to men and younger populations. Furthermore, the age range overlaps substantially with the perimenopausal and menopausal period, during which hormonal changes can independently influence sleep, autonomic function, and fatigue, representing a potential uncontrolled confounder. Participants were also recruited through support groups and patient and public involvement and engagement activities rather than clinician-confirmed diagnosis, which may have introduced heterogeneity in the sample. In addition, the absence of a healthy control group limits conclusions about whether the observed relationships are specific to long COVID–related fatigue.

Second, several methodological limitations should be acknowledged. HRV was derived from a consumer-grade Fitbit Inspire 3 using proprietary photoplethysmography algorithms, and spectral HRV measures, particularly LF power and the LF/HF ratio, have shown limited agreement with ECG-based measurements in previous validation studies. Consequently, these measures should be interpreted as indicative rather than definitive markers of autonomic function. The study also did not control for important factors known to influence HRV, including caffeine, alcohol, medications, or undiagnosed sleep disorders such as obstructive sleep apnea. Symptom reports were completed at participants’ convenience rather than at fixed times, introducing potential recall bias and variability in the temporal alignment between symptoms, activity, and sleep. Additionally, excluding nights with poor HRV coverage (<50%) may have disproportionately removed periods of disturbed sleep, potentially introducing selection bias.

Finally, limitations related to outcome measurement and statistical modeling should be considered. Fatigue ratings were heavily clustered around moderate severity, reducing variability and potentially limiting the ability to detect day-to-day associations; future studies would benefit from validated multidimensional fatigue measures. Although the reverse-direction model showed acceptable improvement following log transformation, residuals remained imperfectly normal, indicating that model assumptions were only approximately satisfied.

Fatigue was modeled as a numeric outcome in the Sleep × Steps interaction model, whereas it is treated as ordinal in the primary cumulative link mixed models; because the observed fatigue scale has only three levels (mild, moderate, severe) with no assumed interval spacing, this continuous treatment is a simplification, and the interaction estimate should be interpreted with corresponding caution. In addition, because step counts were accumulated between sleep offset and a variable symptom report time, total steps are correlated with time awake at the point of reporting, and the Sleep × Steps interaction could therefore partly reflect a Sleep × Time Awake interaction.

### Clinical Implications and Future Directions

These exploratory findings generate several hypotheses for future investigation. (1) Sleep duration and REM sleep are within-person day-to-day predictors of fatigue (the third association surviving correction, between sleep efficiency and next-day energy, is not carried forward here as it most plausibly reflects chance); sleep hygiene interventions targeting these metrics may offer symptom benefit. (2) HRV-RMSSD is a between-person correlate of average symptom burden, positioning it as a potential baseline biomarker rather than a real-time tracking tool. Whether autonomic-targeted interventions (HRV biofeedback, vagus nerve stimulation, slow breathing, mind-body practices) can improve between-person HRV levels and thereby reduce symptom burden in long COVID warrants experimental investigation [42,43]. (3) The preliminary, replication-dependent evening energy → nocturnal HRV association raises the hypothesis not yet supported by corrected evidence that evening relaxation strategies targeting subjective energy may support nocturnal autonomic recovery; testing this requires randomized experimental designs. (4) Activity pacing should incorporate recent sleep quality, with higher-exertion activities potentially better tolerated following nights of adequate sleep.

Randomized controlled trials manipulating sleep opportunity, HRV via biofeedback, or relaxation before sleep with crossover designs within the same individuals to leverage within-person variation would be the appropriate next step to test these hypotheses.

### Conclusions

This exploratory longitudinal study identified bidirectional, temporally specific associations between sleep, nocturnal autonomic function, and daily symptoms in individuals with long COVID. Within-person analyses revealed that sleep duration and REM sleep predict day-to-day fatigue fluctuations, while HRV-RMSSD differentiated individuals with better versus worse average symptom burden (a between-person rather than day-to-day predictor). A third association, between sleep efficiency and next-day energy, also survived correction but ran counter to expectation and most plausibly reflects chance. Evening energy, but not fatigue, showed a within-person association with subsequent nocturnal autonomic function. These findings underscore the importance of disaggregating within-person from between-person effects in ecological momentary assessment research and highlight the potential complementary roles of sleep tracking and HRV monitoring: the former for real-time daily prediction, the latter for characterizing physiological reserve in long COVID management. Experimental work is needed to clarify causal mechanisms.

## Supplementary material

10.2196/99630 Multimedia Appendix 1 Supplementary tables S1-S3—standard cumulative link mixed model results (sensitivity analysis) and pooled Spearman correlations.

## Abbreviations

CLMM: cumulative link mixed model
ECG: electrocardiogram
HF: high-frequency
HRV: heart rate variability
ICC: intraclass correlation coefficient
LF: low-frequency
PEM: postexertional malaise
REM: rapid eye movement
rmcorr: repeated-measures correlation
RMSSD: root mean square of successive differences
VIF: variance inflation factor

## References

[1] Davis HE, Assaf GS, McCorkell L (2021). Characterizing long COVID in an international cohort: 7 months of symptoms and their impact. EClinicalMedicine.

[2] Nalbandian A, Sehgal K, Gupta A (2021). Post-acute COVID-19 syndrome. Nat Med.

[3] Huang C, Huang L, Wang Y (2021). 6-month consequences of COVID-19 in patients discharged from hospital: a cohort study. Lancet.

[4] Mehandru S, Merad M (2022). Pathological sequelae of long-haul COVID. Nat Immunol.

[5] Gorna R, MacDermott N, Rayner C (2021). Long COVID guidelines need to reflect lived experience. Lancet.

[6] Wong TL, Weitzer DJ (2021). Long COVID and myalgic encephalomyelitis/chronic fatigue syndrome (ME/CFS)-a systematic review and comparison of clinical presentation and symptomatology. Medicina (Kaunas).

[7] Asarcikli LD, Hayiroglu Mİ, Osken A, Keskin K, Kolak Z, Aksu T (2022). Heart rate variability and cardiac autonomic functions in post-COVID period. J Interv Card Electrophysiol.

[8] Barizien N, Le Guen M, Russel S, Touche P, Huang F, Vallée A (2021). Clinical characterization of dysautonomia in long COVID-19 patients. Sci Rep.

[9] Hasty F, García G, Dávila H, Wittels SH, Hendricks S, Chong S (2021). Heart rate variability as a possible predictive marker for acute inflammatory response in COVID-19 patients. Mil Med.

[10] Mooren FC, Böckelmann I, Waranski M (2023). Autonomic dysregulation in long-term patients suffering from post-COVID-19 syndrome assessed by heart rate variability. Sci Rep.

[11] Chouchou F, Desseilles M (2014). Heart rate variability: a tool to explore the sleeping brain?. Front Neurosci.

[12] (1996). Heart rate variability: standards of measurement, physiological interpretation and clinical use. Task Force of the European Society of Cardiology and the North American Society of Pacing and Electrophysiology. Circulation.

[13] Billman GE (2013). The LF/HF ratio does not accurately measure cardiac sympatho-vagal balance. Front Physio.

[14] Tobaldini E, Nobili L, Strada S, Casali KR, Braghiroli A, Montano N (2013). Heart rate variability in normal and pathological sleep. Front Physiol.

[15] Aboagye NY, Germann M, Baker KF, Baker MR, Del Din S (2025). Feasibility of predicting next-day fatigue levels using heart rate variability and activity-sleep metrics in people with post-COVID fatigue. Front Digit Health.

[16] Vivaldi G, Talaei M, Blaikley J (2025). Bidirectional relationship between sleep problems and long COVID: a longitudinal analysis of data from the COVIDENCE UK study. BMJ Open Resp Res.

[17] Board on the Health of Select Populations, Institute of Medicine, Committee on the Diagnostic Criteria for Myalgic Encephalomyelitis/Chronic Fatigue Syndrome (2015). Beyond Myalgic Encephalomyelitis/Chronic Fatigue Syndrome: Redefining an Illness.

[18] Twomey R, DeMars J, Franklin K, Culos-Reed SN, Weatherald J, Wrightson JG (2022). Chronic fatigue and postexertional malaise in people living with long COVID: an observational study. Phys Ther.

[19] Dehlia A, Guthridge MA (2024). The persistence of myalgic encephalomyelitis/chronic fatigue syndrome (ME/CFS) after SARS-CoV-2 infection: a systematic review and meta-analysis. J Infect.

[20] Müller P, Achraf A, Zou L, Apfelbacher C, Erickson KI, Müller NG (2020). COVID‐19, physical (in‐)activity, and dementia prevention. Alzheimers Dement (N Y).

[21] Jin X, Chen Y, Feng H (2024). Association of accelerometer-measured sleep duration and different intensities of physical activity with incident type 2 diabetes in a population-based cohort study. J Sport Health Sci.

[22] Percutaneous auricular nerve stimulation for treating post-COVID fatigue (Pausing-PCF). COVID fatigue.

[23] Higuchi D, Takahashi Y, Tanaka S, Tomita Y (2025). Validation of heart rate measured by the consumer-level wearable device Fitbit: a cross-sectional observational study. J Phys Ther Sci.

[24] Bent B, Goldstein BA, Kibbe WA, Dunn JP (2020). Investigating sources of inaccuracy in wearable optical heart rate sensors. NPJ Digit Med.

[25] Giurgiu M, von Haaren-Mack B, Fiedler J (2025). The wearable landscape: issues pertaining to the validation of the measurement of 24-h physical activity, sedentary, and sleep behavior assessment. J Sport Health Sci.

[26] Cao R, Azimi I, Sarhaddi F (2022). Accuracy assessment of Oura Ring nocturnal heart rate and heart rate variability in comparison with electrocardiography in time and frequency domains: comprehensive analysis. J Med Internet Res.

[27] Sarhaddi F, Kazemi K, Azimi I (2022). A comprehensive accuracy assessment of Samsung smartwatch heart rate and heart rate variability. PLoS One.

[28] Heart rate time series. developer.

[29] Haghayegh S, Khoshnevis S, Smolensky MH, Diller KR, Castriotta RJ (2019). Accuracy of wristband Fitbit models in assessing sleep: systematic review and meta-analysis. J Med Internet Res.

[30] Shaffer F, McCraty R, Zerr CL (2014). A healthy heart is not a metronome: an integrative review of the heart’s anatomy and heart rate variability. Front Psychol.

[31] FatigueSense.

[32] Bakdash JZ, Marusich LR (2017). Repeated measures correlation. Front Psychol.

[33] Mundlak Y (1978). On the pooling of time series and cross section data. Econometrica.

[34] Dani M, Dirksen A, Taraborrelli P (2021). Autonomic dysfunction in ‘long COVID’: rationale, physiology and management strategies. Clin Med (Lond).

[35] Tabacof L, Wood J, Breyman E (2023). Dysautonomia, but not cardiac dysfunction, is common in a cohort of individuals with long COVID. J Pers Med.

[36] Vernon SD, Hartle M, Sullivan K (2023). Post-exertional malaise among people with long COVID compared to myalgic encephalomyelitis/chronic fatigue syndrome (ME/CFS). Work.

[37] Maltagliati S, Fessler L, Yu Q (2025). Effort minimization: a permanent, dynamic, and surmountable influence on physical activity. J Sport Health Sci.

[38] Zou L, Sasaki JE, Wei GX (2018). Effects of mind-body exercises (Tai Chi/yoga) on heart rate variability parameters and perceived stress: a systematic review with meta-analysis of randomized controlled trials. J Clin Med.

[39] Zou L, SasaKi JE, Wang H, Xiao Z, Fang Q, Zhang M (2017). A systematic review and meta-analysis of Baduanjin qigong for health benefits: randomized controlled trials. Evid Based Complement Alternat Med.

[40] Liu J, Yang Y, Li C (2024). Effects of mind-body qigong exercise on overall health, fatigue/sleep, and cognition in older Chinese immigrants in the US: an intervention study with control. J Aging Res.

[41] White PD, Goldsmith KA, Johnson AL (2011). Comparison of adaptive pacing therapy, cognitive behaviour therapy, graded exercise therapy, and specialist medical care for chronic fatigue syndrome (PACE): a randomised trial. Lancet.

[42] Lehrer P, Kaur K, Sharma A (2020). Heart rate variability biofeedback improves emotional and physical health and performance: a systematic review and meta analysis. Appl Psychophysiol Biofeedback.

[43] Bonaz B, Sinniger V, Pellissier S (2016). Vagal tone: effects on sensitivity, motility, and inflammation. Neurogastroenterol Motil.

