# Supplementary material for: Bidirectional Associations Between Autonomic Function, Sleep Quality, and Daily Fatigue and Energy Symptoms in Long COVID: Longitudinal Digital Health Study
Source: JMIR Cardio. 2026 Sep 14;10:e99630. doi: 10.2196/99630 (PMC13574307; doi:10.2196/99630)
Supplement: Supplementary file 1 [file cardio-v10-e99630-s001.docx]

*Multimedia Appendix 1 to: Bidirectional Associations Between Autonomic Function, Sleep Quality, and Daily Fatigue and Energy Symptoms in Long COVID: A Longitudinal Digital Health Study*

## Multimedia Appendix 1

**Supplementary Tables S1–S3: Standard CLMM Results (Sensitivity Analysis) and Pooled Spearman Correlations**

### Table S1. Parsimonious CLMMs: Sleep Duration + HRV-RMSSD + LF/HF Ratio

| Model | Predictor | β (SE) | z | p-value | OR | 95% CI (OR) |
| --- | --- | --- | --- | --- | --- | --- |
| Fatigue (n=215) | Sleep Duration | −0.702 (0.177) | −3.98 | <.001 | 0.50 | [0.35, 0.70] |
|  | HRV-RMSSD | −0.492 (0.247) | −1.99 | .05 | 0.61 | [0.38, 1.00] |
|  | LF/HF Ratio | −0.063 (0.286) | −0.22 | .82 | 0.94 | [0.54, 1.65] |
| Energy (n=214) | Sleep Duration | +0.451 (0.164) | +2.75 | .006 | 1.57 | [1.14, 2.16] |
|  | HRV-RMSSD | +0.613 (0.236) | +2.60 | .009 | 1.85 | [1.17, 2.93] |
|  | LF/HF Ratio | +0.007 (0.299) | +0.02 | .98 | 1.01 | [0.56, 1.81] |

### Table S2. Comprehensive CLMMs: All Sleep Metrics + HRV-RMSSD + LF/HF Ratio

| Model | Predictor | β (SE) | z | p-value | OR | 95% CI (OR) |
| --- | --- | --- | --- | --- | --- | --- |
| Fatigue (n=211) | Sleep Duration | −0.380 (0.232) | −1.64 | .10 | 0.68 | [0.43, 1.08] |
|  | Sleep Efficiency | +0.075 (0.164) | +0.46 | .65 | 1.08 | [0.78, 1.49] |
|  | Deep Sleep | −0.133 (0.181) | −0.74 | .46 | 0.88 | [0.61, 1.25] |
|  | REM Sleep | −0.376 (0.239) | −1.57 | .12 | 0.69 | [0.43, 1.10] |
|  | HRV-RMSSD | −0.705 (0.316) | −2.23 | .03 | 0.49 | [0.26, 0.92] |
|  | LF/HF Ratio | −0.076 (0.277) | −0.27 | .79 | 0.93 | [0.54, 1.60] |
| Energy (n=210) | Sleep Duration | +0.311 (0.222) | +1.40 | .16 | 1.36 | [0.88, 2.11] |
|  | Sleep Efficiency | −0.016 (0.171) | −0.10 | .92 | 0.98 | [0.70, 1.38] |
|  | Deep Sleep | +0.168 (0.189) | +0.89 | .37 | 1.18 | [0.82, 1.71] |
|  | REM Sleep | +0.052 (0.235) | +0.22 | .83 | 1.05 | [0.66, 1.67] |
|  | HRV-RMSSD | +0.546 (0.225) | +2.42 | .02 | 1.73 | [1.11, 2.69] |
|  | LF/HF Ratio | +0.007 (0.311) | +0.02 | .98 | 1.01 | [0.55, 1.85] |

Note: Odds ratios and 95% confidence intervals are reported for every predictor (significant and non-significant). These standard CLMMs do not disaggregate within- from between-person effects; with energy ICC=0.491, HRV-RMSSD estimates in standard models reflect a mixture of both. The Mundlak models (Table 4) are the primary analysis. VIFs for all predictors ranged 1.10–1.84 (no collinearity concern). Random intercept models; random slopes not warranted (LR tests: fatigue p=.52, energy p=.87). Because these models include only 14 level-2 units (participants), model-based standard errors may be anticonservative and the confidence intervals and p-values should be interpreted with corresponding caution. A small-sample degrees-of-freedom correction was considered but not applied, as no standard implementation is available for cumulative link mixed models; this is acknowledged as a limitation.

### Table S3. Pooled Spearman Correlations (Exploratory; Not Corrected for Within-Person Clustering)

These are presented for descriptive comparison only. Pooled correlations treat 260 repeated observations from 14 participants as independent and will overestimate associations inflated by between-person differences (particularly for energy, ICC=0.491). Primary bivariate results are the rmcorr estimates in Table 3.

| Predictor | Fatigue ρ (p-value) | N | Energy ρ (p-value) | N |
| --- | --- | --- | --- | --- |
| Sleep Duration | −0.145 (.03) | 215 | +0.013 (.85) | 214 |
| Sleep Efficiency | +0.053 (.44) | 215 | −0.099 (.15) | 214 |
| Deep Sleep | −0.162 (.02) | 211 | +0.041 (.55) | 210 |
| REM Sleep | −0.271 (<.001) | 213 | +0.142 (.04) | 212 |
| HRV-RMSSD | −0.317 (<.001) | 222 | +0.416 (<.001) | 221 |
| HRV-HF | −0.359 (<.001) | 222 | +0.498 (<.001) | 221 |
| HRV-LF | −0.280 (<.001) | 222 | +0.286 (<.001) | 221 |
| LF/HF Ratio | +0.204 (.002) | 222 | −0.422 (<.001) | 221 |
| Daily Steps | −0.056 (.41) | 222 | +0.006 (.93) | 221 |

Note: These are uncorrected p-values from pooled Spearman correlations. Not adjusted for multiple comparisons. N refers to observations (repeated measures within 14 participants).
